# Three-dimensional culture of human meniscal cells: Extracellular matrix and proteoglycan production

**DOI:** 10.1186/1472-6750-8-54

**Published:** 2008-06-26

**Authors:** Helen E Gruber, David Mauerhan, Yin Chow, Jane A Ingram, H James Norton, Edward N Hanley, Yubo Sun

**Affiliations:** 1 Department of Orthopaedic Surgery, Carolinas Medical Center, Charlotte, NC, USA; 2 Department of Biostatistics, Carolinas Medical Center, Charlotte, NC, USA

## Abstract

**Background:**

The meniscus is a complex tissue whose cell biology has only recently begun to be explored. Published models rely upon initial culture in the presence of added growth factors. The aim of this study was to test a three-dimensional (3D) collagen sponge microenvironment (without added growth factors) for its ability to provide a microenvironment supportive for meniscal cell extracellular matrix (ECM) production, and to test the responsiveness of cells cultured in this manner to transforming growth factor-β (TGF-β).

**Methods:**

Experimental studies were approved prospectively by the authors' Human Subjects Institutional Review Board. Human meniscal cells were isolated from surgical specimens, established in monolayer culture, seeded into a 3D scaffold, and cell morphology and extracellular matrix components (ECM) evaluated either under control condition or with addition of TGF-β. Outcome variables were evaluation of cultured cell morphology, quantitative measurement of total sulfated proteoglycan production, and immunohistochemical study of the ECM components chondroitin sulfate, keratan sulfate, and types I and II collagen.

**Result and Conclusion:**

Meniscal cells attached well within the 3D microenvironment and expanded with culture time. The 3D microenvironment was permissive for production of chondroitin sulfate, types I and II collagen, and to a lesser degree keratan sulfate. This microenvironment was also permissive for growth factor responsiveness, as indicated by a significant increase in proteoglycan production when cells were exposed to TGF-β (2.48 μg/ml ± 1.00, mean ± S.D., vs control levels of 1.58 ± 0.79, p < 0.0001). Knowledge of how culture microenvironments influence meniscal cell ECM production is important; the collagen sponge culture methodology provides a useful in vitro tool for study of meniscal cell biology.

## Background

The meniscus of the knee is a complex dense fibrous biologic structure with a design to provide both stability and a shock-absorber function. Collagenous components of the extracellular matrix (ECM) help provide tensile strength, and proteoglycan ECM components contribute to the shock absorber function. The meniscus contains both a vascularized outer portion and an inner, avascular region.

Appropriate function of the menisci are needed for normal knee biomechanical function. Aging and degeneration of the menisci result in tears being a common knee injury; 15% of all sports knee injuries involve the meniscus [1]. Initial meniscal injury causes patient pain and disability, and further meniscal damage and/or loss is associated with degenerative joint changes ultimately leading to osteoarthritis [2-4]. In spite of patient pain and major health care costs involved with meniscal damage, research on the biology of the meniscal cells and the potential for tissue engineering applications for the meniscus are relatively new fields of endeavor [5].

Arnoczky has summarized major issues related to meniscal repair using scaffolds and cells [6], noting that although some preliminary data had been done using meniscal fibrochondrocytes, fibroblasts, or synovial cells to produce a fibrocartilaginous tissue, additional studies were needed to characterize the cellular features and ECM produced. Several of the biologic considerations involved in creating a tissue engineered meniscus have also been described by Arnoczky [7], who has summarized several of the important cell carrier (or scaffold) features, including support of cell proliferation and ECM production, diffusion of nutrients, possible use as a carrier for cytokines, and biomechanical considerations. The latter has been addressed also by Setton et al [8]. Cell carrier materials should also degrade at a rate similar to the cell deposition rate of the ECM [9].

Tissue engineering approaches include the use of cells in specific cell carrier constructs or scaffolds which can provide specific cellular microenvironments directing desired cellular activities, including ECM production, cell proliferation and activation of cell signaling pathways [10,11]. or allow delivery of bioactive molecules, proteins or drugs [12,13].

In the present study, we primarily focus upon use of a collagen sponge scaffold for 3D human meniscal cell culture. Rodkey has illustrated previous use of a bovine collage meniscus scaffold implanted in the medial meniscus of the dog [7]. Our laboratory has also previously used such a construct in autologous disc cell implantation in a small rodent model and in other studies evaluating ECM deposition by human disc cells [14-16].

In this work, we have a primary interest in the ability of cultured human meniscus cells to attach and expand within this carrier, and to produce collagens and proteoglycans between and around these 3D cultured cells. Meniscal cell responsiveness to TGF-β was also investigated.

## Results

**Figure 1:**
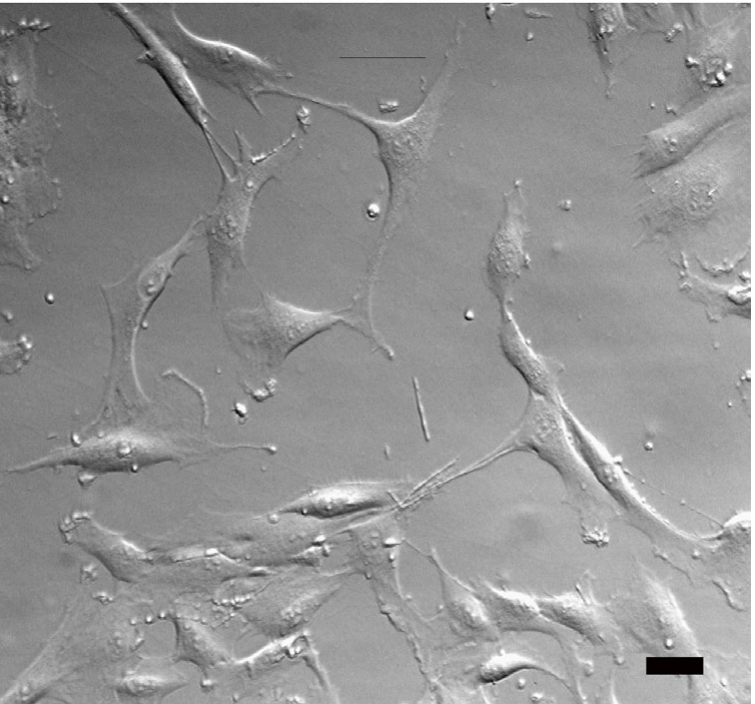
**Low confluence human meniscus cells are spindle-shaped in monolayer culture.** Note long processes which extend from the cells. (Hoffman modulation contrast image; bar = 10 μm).

Meniscal cells grew well from the initial explant culture and expanded well in monolayer culture. As shown in Figure 1, cells in early (non-confluent) stages had a fibroblast-like morphology in monolayer, with prominent long cell processes extending from the cells.

**Figure 2:**
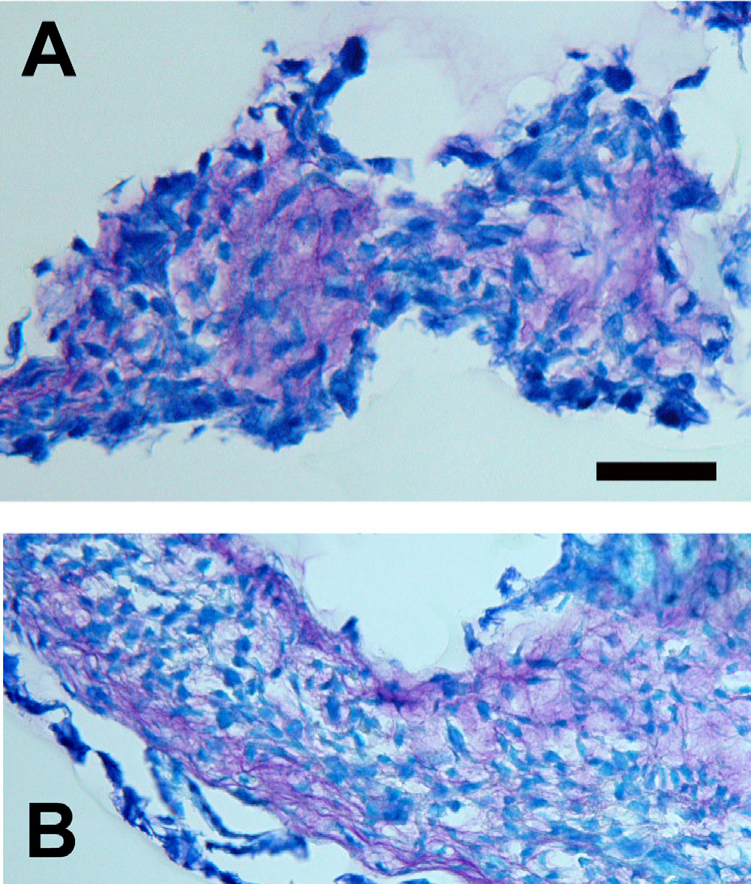
**Histologic images of two micromass cultures formed by human meniscus cells.** Note the presence of ECM rich in proteoglycans as indicated by the pink stain. (Paraffin-embedded micromass nodule, stained with toluidine blue; bar = 50 μm).

An initial series of experiments assessed the utility of the micromass culture method with human meniscal cells. Figure 2 shows that the resulting cell pellet contained abundant proteoglycans; proteoglycans are stained pink in this figure. However, these micromass cultures were delicate, technically difficult to feed and maintain, and resulted in small specimens which were challenging to examine with histologic methods. Therefore, we next investigated a collagen sponge 3D microenvironment for meniscal cell culture.

Meniscal cells were studied from 11 subjects to investigate cell behavior, ECM formation and to quantify proteoglycan production in 3D collagen sponge culture. Specimens of meniscal cells in this 3D construct were easily fed, handled and processed for histologic examination. The study population contained 7 males and 4 females; 7 medial and 7 lateral meniscal cultures were assessed. Mean age for the medial meniscal cell surgical patients was 65.2 years + 10.9 (7) (mean ± S.D. (n)), and for the lateral meniscal cell surgical patients 67.2 + 6.5 (7). Mean meniscal grades did not differ between the lateral and medial groups (3.5 ± 0.7 (7) vs 3.4 ± 0.5 (7), respectively).

Proteoglycan production in 3D culture over 14 days was assessed with the DMB assay. Total proteoglycan values (μg/ml ECM digest) did not differ between the lateral and medial study populations (2.097 ± 0.214 (7) vs 2.369 ± 0.272 (7), respectively).

**Figure 3:**
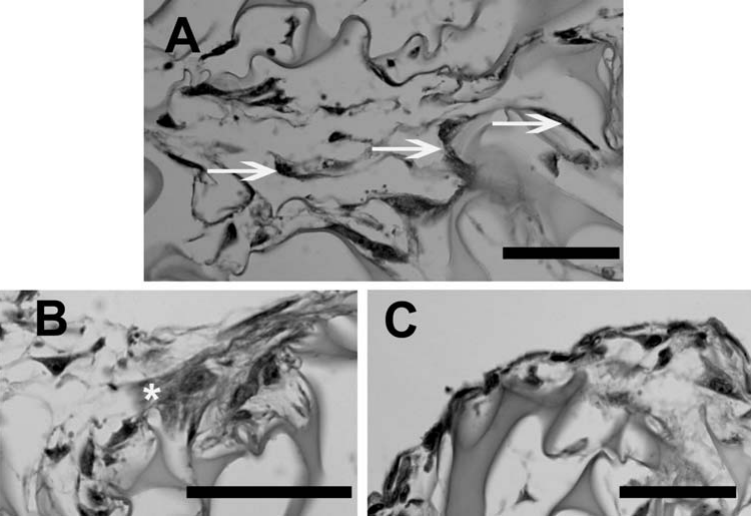
**Histologic images of human meniscus cells cultured under control conditions in the 3D collagen sponge.** A. Arrows show good attachment of cells to the sponge surface. B. * denotes concentration of ECM. C. Margin shows a tight layer of cells along the perimeter of the sponge. (Paraffin embedded toluidine-blue stained specimens; for all images, bar = 50 μm).

Meniscal cells attached well to the 3D sponge surface as shown in Figure 3A. ECM produced by cells filled cavities within the 3D sponge matrix and was present between and around cells (Figure 3B). Along the margin of the sponge, at the sponge-media interface, cells formed a boundary layer which often consisted of several layers of cells (Figure 3C).

We next examined the response of human meniscal cells in 3D sponge culture during exposure to 5 ng/ml TGF-β. This portion of our study utilized meniscal cells derived form 7 lateral and 8 medial menisci from 9 subjects (8 females, 1 male). Mean subject age was 64.6 years ± 8.5 (9). Cells were derived from three grade 3 menisci, and 12 grade 4 menisci. Results were analyzed in terms of quantitative assessment of total proteoglycan production by control vs TGF-β-treated cells, and by immunohistologic examination of ECM components.

**Figure 4:**
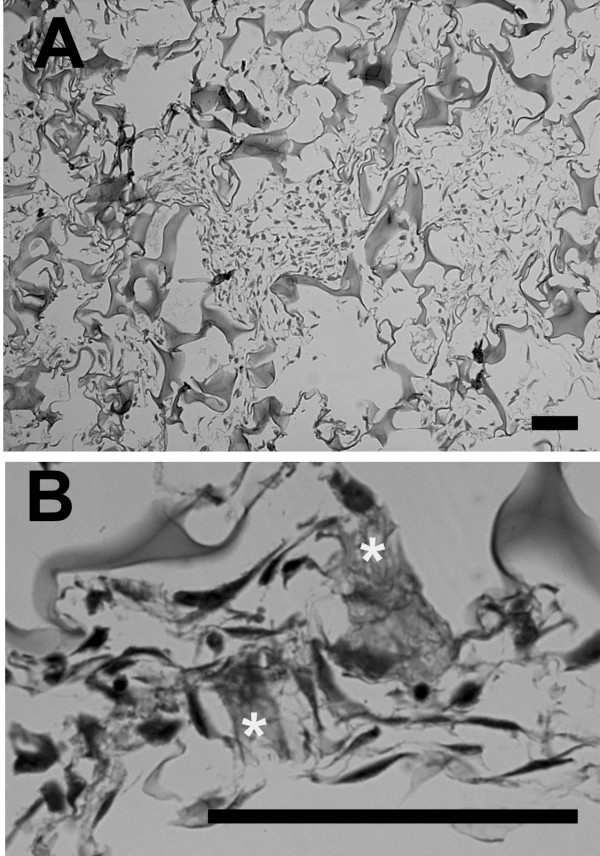
**Histologic features of human meniscal cells cultured in 3D collagen sponge in the presence of TGF-β.** A, note the high cell density; B, note accumulations of ECM marked by *. (Paraffin embedded sections stained with toluidine blue; for both images, bar = 50 μm).

Exposure to TGF-β significantly increased proteoglycan production by cells in 3D sponge culture. TGF-β-treated cells had a mean proteoglycan level of 2.48 ± 1.00 (15), (mean ± S.D. (n), whereas the mean paired control level was 1.58 ± 0.79 (15), p < 0.0001. Cells cultured in the presence of TGF-β showed normal morphology and good proliferation (Figure 4A and 4B).

**Figure 5:**
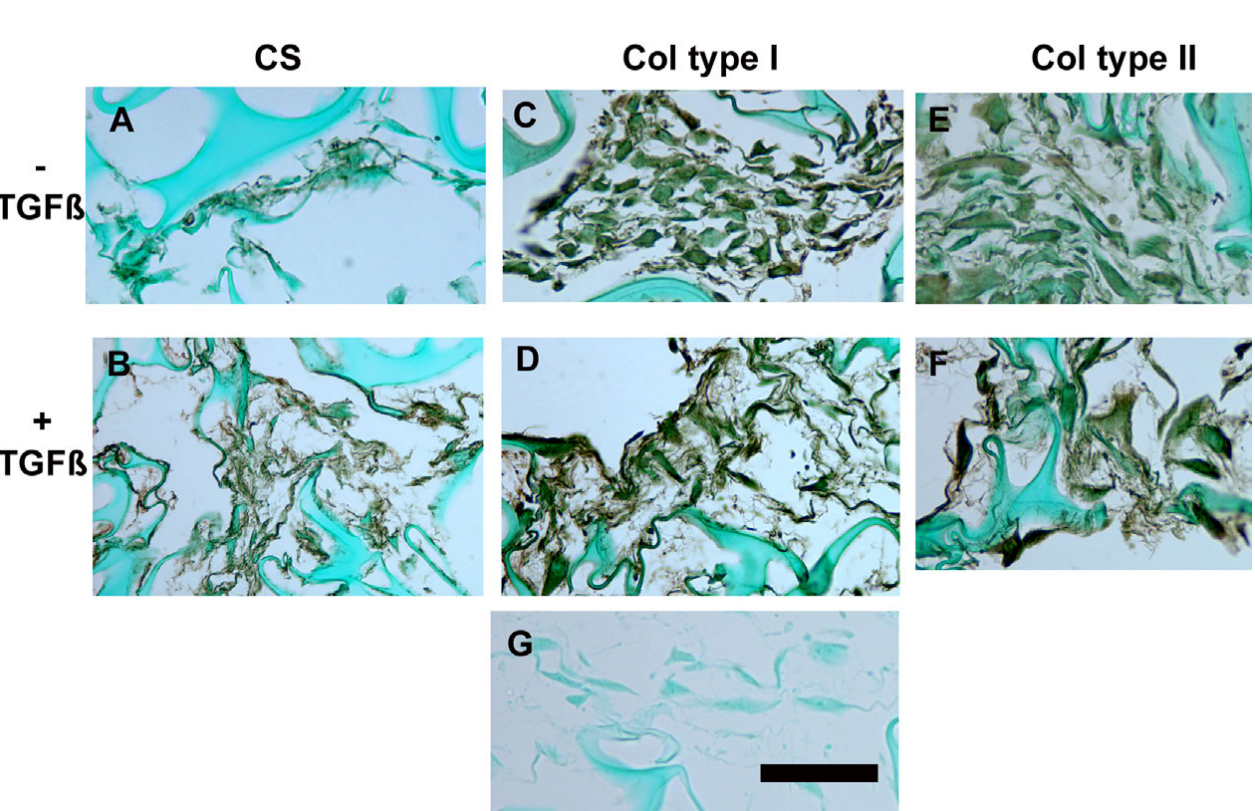
**Immunolocalization of major ECM components in control and TGF-β-treated meniscal cells in 3D culture.** The top row presents images from control cells; the second row presents images for TGF-β-treated cells. Chondroitin sulfate (CS): A: control cells show modest CS content in the ECM. B: ECM formed by TGF-β-treated cells shows more abundant CS compared to controls. Type I collagen (Col type I) is abundant in both ECM formed by control (C) and TGF-β-treated cells (D). Type II collagen (Col type II): Control cells produced less type II collagen (E) when compared to ECM from TGF-β-treated cells (F). Figure 5G presents a representative negative control specimen. (For all images, bar in G = 50 μm).

Immunohistochemical studies were performed to evaluate in vitro 3D production of selected major ECM components: keratan sulfate, chondroitin sulfate, and types I and II collagens. For keratan sulfate, localization was primarily cytoplasmic, with scant keratan present in the ECM (data not shown). In contrast, chondroitin sulfate was abundant in ECM surrounding cells in both control and TGF-β-treated meniscal cells (Figure 5A and 5B). Abundant type I collagen was seen in ECM under control culture conditions (Figure 5C); type I production was also extensive in the ECM of TGF-β-treated meniscal cells (Figure 5D). Although control cultures produced substantial amounts of type II collagen (Figure 5E), ECM formed by TGF-β-treated cells appeared to have qualitatively greater amounts (Figure 5F).

## Discussion

In this study we have shown that cells from even lower grades of menisci with greater degeneration can be established in culture, expanded and passaged, and studied in 3D culture microenvironments. It is interesting to note that meniscal cells derived from one of our study subjects with a grade 0 meniscus (i.e. a healthy meniscus) yielded a proteoglycan production value not different from the study population means for the lateral and medial cells (2.421 μg/ml compared to the lateral mean of 2.097 and medial mean of 2.369). We have also tested cells from a 12 year old child with an osteosarcoma, again with a meniscal grade 0; this individuals proteoglycan production level in 3D culture was also similar to the study means (2.758). Thus, although our study has the shortcoming of utilizing primarily more degenerated menisci with grades 3–5, we have a small body of evidence that the 3D collagen sponge methodology works well with meniscal cells from both health and degenerated menisci.

Although several other recent tissue culture studies that have utilized other 3D methodologies, several have features which make those methods more difficult to use in experimental studies. Adesida et al. utilized a micromass-like methodology, but this required that the culture be carried out with supplemented chondrogenic factors (insulin, transferrin, selenite, dexamethasone, TGF-β) [17,18]. Such media additives should be kept in mind when evaluating the resulting cell phenotype.

Other meniscal cell culture methodologies include the gelatin hydrogel system utilized by Ishida et al. [19]and polyglycolic acid scaffolds as utilized by Stewart et al. [20], Marsano et al. [21], and Kang et al. [22]. The polyclycolic carrier systems are complicated by the acidic microenvironment associated with their degradation, and long-lasting presence of macrophages/giant cells during this process [23]. Previous work by Verdonk et al. utilized a more traditional alginate culture methodology, but histologic illustrations showed little ECM surrounding cells [24].

Our study presents data on a relatively small patient population. It is important that future additional work address cells derived from both medial and lateral meniscal sites, and that studies measure proteoglycan production at additional time points which will expand our data from 7 days of culture in micromass and 14 days of culture in 3D collagen sponges.

Because of the clinical importance of meniscal tears and meniscal degeneration, we hope that culture methods such as those described here will be used in future studies of meniscal cell gene expression and tissue engineering applications.

## Conclusion

The present findings on ECM production under control conditions by human meniscal cells provides an important advance over routine monolayer cell culture. The 3D microenvironment provides cues to the cells in a fashion that is more like the in vivo condition of cells. Our studies here utilized 20% fetal bovine serum to accelerate proliferation and thus lessen the time cells were kept in monolayer culture prior to experimental use. Although micromass culture did show ECM production, this technique is extremely tedious and limited in analytical studies. The collagen sponge, in contrast, is easily manipulated, does not require use of specialized chondrogenic media to allow ECM production, provides ample room for meniscal cell expansion and ECM production, and is easily embedded for morphologic studies.

## Methods

### (i) Clinical Study Population

Experimental studies were approved prospectively by the authors' Human Subjects Institutional Review Board. Meniscal specimens were obtained from osteoarthritis patients undergoing total joint replacement surgery and transported immediately to the laboratory from the surgical suite. All the meniscal specimens obtained from osteoarthritis patients displayed clear signs of severe fibrillation and tears (grades 3 and 4). One meniscal specimen with normal-appearance (grade 0) was used in this study from a patient undergoing meniscectomy due to injury caused by an auto accident. Menisci were scored according to the following scale: grade 0, normal; grade 1, minimal fibrillation; grade 2, moderate fibrillation but without tears; grade 3, severe fibrillation and the presence of incomplete tears; grade 4, severe fibrillation with complete tears or multiple incomplete tears.

### (ii) Monolayer cell culture

Meniscal cells were isolated from surgical specimens as previously described for the intervertebral disc using explant techniques [25] and expanded in monolayer culture for experimental use. Cells were cultured in sterile modified Minimal Essential Media with Earle's salts (MEM; Gibco, Grand Island, NY) with L-glutamine 1% (v/v) (Irvine Scientific, Santa Ana, CA), nonessential amino acids 1% (v/v) (Irvine Scientific), penicillin-streptomycin 1% (Irvine Scientific) and fetal bovine serum 20% (Gibco). All cultures were grown at 37°C under conditions of 95% relative humidity and 5% CO_2_. Cells were fed with fresh media 3 times a week.

### (iii) Standardized Design for 3D Sponge Cell Culture Experiments

Cells were P2 or 3 when used in experiments. Sterile Gelfoam^® ^(Pharmacia & Upjohn Co., Kalamazoo, MI) sponges, an absorbable gelatin sponge prepared from purified porcine skins gelatin USP granules, were trimmed into 0.5 cm^3 ^cubes. Following preliminary studies testing cell concentrations, the work reported here used cell suspensions containing an average of 100,000 cells in Minimal Essential Medium with 20% fetal bovine serum (MEM20); this concentration was injected into each sponge cube with a pipette. Replicate collagen sponges were placed on Costar Transwell Clear Inserts (Costar) in 24-well plates and soaked with 2 ml MEM20. Seeded constructs were placed in the incubator (37°C, 5% CO_2_, 95% humidity) and fed with fresh media 3 times a week. The TGF-β studies were carried out on cells cultured in 3D for 14 days with or without the addition of 5 ng/ml TGF-β (Sigma, St. Louis, MO). At the end of the 14 day period, replicate specimens were assessed either with histology and immunohistochemistry or for quantitative analysis of proteoglycan content (see below).

### (iv) Micromass 3D Culture

Meniscal cells were also grown in micromass culture using modifications of Tare et al [26] and Paulsen and Solursh [27]. 200,000 cells were used to form each micromass culture. Micromass cultures were fed as described above and terminated after 7 days of culture. Upon termination, the micromass cultures were carefully transferred to a microcentrifuge tube, spun, and embedded in 1% sea plaque agarose to facilitate subsequent specimen handling. Micromasses were fixed in 10% neutral buffered formalin for 1 hour, placed in 70% ethanol, and processed for paraffin embedding.

### (v) Morphologic and Immunohistochemical Analyses

At termination of culture, specimens were fixed in 10% neutral buffered formalin (Allegiance, McGaw Park, IL) for one hour and then transferred to 70% ethyl alcohol (AAPER, Shelbyville, KY) and processed in paraffin using a Shandon Pathcentre Automated Tissue Processor (ThermoShandon, Pittsburgh, PA). Specimens were embedded in Paraplast Plus (ThermoShandon) paraffin and 4 μm serial sections were cut with a Leica (Nussloch, Germany) RM2025 microtome. Sections were mounted on Superfrost-Plus microscope slides (Allegiance) and stained with toluidine blue for routine study, or were utilized for immunohistochemistry.

#### Immunohistochemistry

Collagen sponges were bisected and the two halves embedded on edge. Specimens were embedded in Paraplast Plus paraffin (ThermoShandon), and 4 mm serial sections cut with a Leica (Nussloch, Germany) RM2025 microtome and mounted on Superfrost-Plus microscope slides (Allegiance). Immunohistochemical localization of types I and II collagen, chondroitin sulfate, decorin and keratan sulfate utilized antibodies used techniques described previously [15,25]. The following antibodies were used at the indicated concentrations: anti-types I and II collagen (Biodesign International, Kennebunk, ME), 20 μg/ml; anti-keratan sulfate (Seikagaku Corporation, Tokyo, Japan), 5 μg/ml; anti-chondroitin sulfate (ICN Biomedicals, Costa Mesa, CA), 20 μg/ml. Endogenous peroxidase was blocked using 3% H202 (Humco, Texarcana, TX). The secondary antibody was Dako LSAB2 biotinylated Link for HRP/AP for 10 minutes followed by peroxidase-conjugated streptavidin (Dako) for 10 minutes and DAB (Dako) for 5 minutes. Slides were counterstained with light green, dehydrated, cleared and mounted with resinous mounting media. Negative controls consisted of Rabbit IgG (Dako, Carpinteria, CA, for collagen I and II) or mouse IgG (Dako, Carpinteria, CA, for all other antibodies) used at the same concentration as each antibody.

### (vi) Biochemical total glycosaminoglycan determination

The 1,9 dimethylmethylene blue (DMB) assay was utilized to assess S-GAG production with modifications of the previously described method [28]. The DMB (Aldrich, Milwaukee, WI) solution was prepared and stored at room temperature in an amber bottle. Collagen sponge constructs containing cells were rinsed 3 times in Hanks Balanced Salt Solution, HBSS, (Gibco, Carlsbad, CA). Replicates were combined in 1.5 ml microcentrifuge tubes and collagenase type V (Sigma) in HBSS (Gibco) was added to produce a final concentration of 1.0 Units/ml. The microcentrifuge tubes were incubated at 37°C until the sponge was completely digested (15–30 minutes). Samples were assayed in duplicate by mixing 0.1 ml of sample with 1.25 ml DMB solution. For standards, 0 – 7.5 μg chondroitin sulfate (Sigma, St. Louis, MO) was added to 1.25 ml DMB solution. All tubes were incubated at room temperature in the dark for 30 minutes followed by centrifugation at room temperature for 15 minutes at 10,000 rpm. One ml of the supernatant was removed and the absorbency read at 595 nm using a Beckman DU 600 spectrophotometer (Fullerton, CA). Formate buffer, pH 3.1, was used as a blank. Sample concentrations were determined from the standard curve and results expressed as μg S-GAG/ml.

### (vii) Statistical analysis

Statistical analysis utilized standard methods using SAS software (version 8.2; SAS Institute, Cary, NC). Methods used included paired t-tests and Pearson correlation statistics. A p-value of less than 0.05 was considered statistically significant.

## Abbreviations

3D: three-dimensional; ECM: extracellular matrix; TGF-β: transforming growth factor-beta; v: volume; MEM: Minimal Essential Media; DMB: dimethylmethylene blue; GAG: glycosaminoglycan.

## Authors' contributions

HEG: preparation of manuscript, experimental design, interpretation; DM: interpretation and specimen procurement; YC and JAI: laboratory work; ENH: interpretation; YS: specimen procurement, interpretation; HJN, statistical analysis.

## References

[B1] Majewski M, Susanne H, Klaus S (2006). Epidemiology of athletic knee injuries:  A 10-year study.. Knee.

[B2] Wojtys EM, Chan DB (2005). Meniscus structure and function.. AAOS Instructional Course Lectures.

[B3] Berthiaume MJ, Raynauld JP, Martel-Pelletier J, Beaudoin G, Bloch DA, Choquette D, Haraoui B, Altman RD, Hochberg M, Meyer JM, Cline GA, Pelletier JP, Labonté.F. (2005). Meniscal tear and extrusion are strongly associated with progression of symptomatic knee osteoarthritis as assessed by quantitative magnetic resonance imaging.. Ann Rheum Dis.

[B4] Rodkey WG (2000). Basic biology of the meniscus and response to injury.. AAOS Instructional Course Lectures.

[B5] Glowacki J (1901). Engineered cartilage, bone, joints, and menisci - Potential for temporomandibular joint reconstruction. Cells Tissues Organs.

[B6] Arnoczky SP (1999). Breakout session 4:  Meniscus.. Clin Orthopaed Res Res.

[B7] Arnoczky SP (1999). Building a meniscus.  Biologic considerations.. Clin Orthopaed Res Res.

[B8] Setton LA, Guilak F, Hsu EW, Vail TP (1999). Biomechanical factors in tissue engineered meniscal repair. Clin Orthopaed Res Res.

[B9] Freed LE, Martin I, Vunjak-Novakovic G (1999). Frontiers in tissue engineering - In vitro modulation of chondrogenesis. Clin Orthop.

[B10] Ingber D, Bell E (1993). Extracellular matrix, cellular mechanics and tissue engineering.. Tissue Engineering  Current Perspectives.

[B11] Marler JJ, Upton J, Langer R, Vacanti JP (1998). Transplantation of cells in matrices for tissue regeneration. Adv Drug Deliv Rev.

[B12] Lee SH, Shin H (2007). Matrices and scaffolds for delivery of bioactive molecules in bone and cartilage tissue engineering.. Advanced Drug Delivery Rev.

[B13] Kretlow JD, Klouda L, Mikos AG (2007). Injectable matrices and scaffolds for drug delivery in tissue engineering.. Advanced Drug Delivery Rev.

[B14] Gruber HE, Johnson TL, Leslie K, J.A. I, Martin D, Hoelscher G, Banks D, Phieffer L, Coldham G, Hanley ENJ (2002). Autologous intervertebral disc cell implantation:  A model using Psammomys obesus, the sand rat.. Spine.

[B15] Gruber HE, Leslie K, Ingram J, Norton HJ, Hanley ENJ (2004). Cell-based tissue engineering for the intervertebral disc:  In vitro studies of human disc cell gene expression and matrix production within selected cell carriers.. The Spine Journal.

[B16] Gruber HE, Hoelscher GL, Leslie K, Ingram JA, Hanley ENJ (2006). Three-dimensional culture of human disc cells within agarose or a collagen sponge:  assessment of proteoglycan production.. Biomaterials.

[B17] Adesida AB, Grady LM, Khan WS, Hardingham TE (2006). The matrix-forming phenotype of cultured human meniscus cells is enhanced after culture with fibroblast growth factor 2 and is further stimulated by hypoxia.. Arthritis Research & Therapy.

[B18] Adesida AB, Grady LM, Khan WS, Millward-Sadler SJ, Salter DM, Hardingham TE (2007). Human meniscus cells express hypoxia inducible factor-1a and increased SOX9 in response to low oxygen tension in cell aggregate culture.. Arthritis Research & Therapy.

[B19] Ishida K, Kuroda R, Miwa M, Tabata Y, Hokugo A, Kawamoto T, Sasaki K, Doita M, Kurosaka M (2007). The regenerative effects of platelet-rich plasma on meniscal cells in vitro and its in vivo application with biodegradable gelatin hydrogel.. Tissue Engineering.

[B20] Stewart K, Pabbruwe M, Dickinson S, Sims T, Hollander AP, Chaudhuri JB (2007). The effect of growth factor treatment on meniscal chondrocyte proliferation and differentiation on polyglycolic acid scaffolds.. Tissue Engineering.

[B21] Marsano A, Millward-Sadler SJ, Salter DM, Adesida A, Hardinghan T, Tognana E, Kon E, Chiari-Grisar C, Nehrer S, Jakob M, Martin I (2007). Differential cartilaginous tissue formation by human synovial membrane, fat pad, meniscuc cells and articular cartilage.. Osteoarthritis & Cartilage.

[B22] Kang SW, Son SM, Lee JS, Lee ES, Lee KY, Park SG, Park JH, Kim BS (2006). Regeneration of whole meniscus using meniscal cells and polymer scaffolds in a rabbit total meniscectomy model.. J Biomed Mater Res.

[B23] Holder WD, Gruber HE, Moore AL, Culberson CR, Anderson W, Burg KJL, Mooney DJ (1998). Cellular ingrowth and thickness changes in polylactide and polyglycolide matrices implanted subcutaneously in the rat.. J Biomed Mater Res.

[B24] Verdonk PCM, Forsyth RG, Wang J, Almqvist KF, Verdonk R, Veys EM, Vergruggen G (2005). Characterisation of human knee meniscuc cell phenotype.. Osteoarthritis & Cartilage.

[B25] Gruber HE, Fisher EC, Desai B, Stasky AA, Hoelscher G, Hanley EN (1997). Human intervertebral disc cells from the annulus:  Three- dimensional culture in agarose or alginate and responsiveness to TGF-b1. Exp Cell Res.

[B26] Tare RS, Howard D, Pound JC, Roach HI, Oreffo ROC (2005). Tissue engineering strategies for cartilage generation - Micromass and three dimensional cultures using human chondrocytes and a continuous cell line. Biochem Biophys Res Commun.

[B27] Paulsen DF, Solursh M (1988). Microtiter micromass cultures of limb-bud mesenchymal cells.. In Vitro Cell Dev Biol.

[B28] Muller G, Hanschke M (1996). Quantitative and qualitative analyses of proteoglycans in cartilage extracts by precipitation with 1,9-dimethylmethylene blue. Conn Tiss Res.

